# On the diversity, phylogeny and biogeography of cable bacteria

**DOI:** 10.3389/fmicb.2024.1485281

**Published:** 2024-11-19

**Authors:** Philip Ley, Jeanine S. Geelhoed, Diana Vasquez-Cardenas, Filip J. R. Meysman

**Affiliations:** ^1^Geobiology Research Group, Department of Biology, University of Antwerp, Antwerp, Belgium; ^2^Department of Biotechnology, Delft University of Technology, Delft, Netherlands

**Keywords:** cable bacteria, microbial diversity, phylogenetics, 16S rRNA gene, aquatic sediments, biogeography

## Abstract

Cable bacteria have acquired a unique metabolism, which induces long-distance electron transport along their centimeter-long multicellular filaments. At present, cable bacteria are thought to form a monophyletic clade with two described genera. However, their diversity has not been systematically investigated. To investigate the phylogenetic relationships within the cable bacteria clade, 16S rRNA gene sequences were compiled from literature and public databases (SILVA 138 SSU and NCBI GenBank). These were complemented with novel sequences obtained from natural sediment enrichments across a wide range of salinities (2–34). To enable taxonomic resolution at the species level, we designed a procedure to attain full-length 16S rRNA gene sequences from individual cable bacterium filaments using an optimized nested PCR protocol and Sanger sequencing. The final database contained 1,876 long 16S rRNA gene sequences (≥800 bp) originating from 92 aquatic locations, ranging from polar to tropical regions and from intertidal to deep sea sediments. The resulting phylogenetic tree reveals 90 potential species-level clades (based on a delineation value of 98.7% 16S rRNA gene sequence identity) that reside within six genus-level clusters. Hence, the diversity of cable bacteria appears to be substantially larger than the two genera and 13 species that have been officially named up to now. Particularly brackish environments with strong salinity fluctuations, as well as sediments with low free sulfide concentrations and deep sea sediments harbor a large pool of novel and undescribed cable bacteria taxa.

## 1 Introduction

Cable bacteria are long, multicellular, filamentous bacteria that occur globally in a wide range of natural sediments (Burdorf et al., 2017), including marine environments, such as salt marshes (Malkin et al., 2014), mangroves (Burdorf et al., 2016), bivalve reefs (Malkin et al., 2017), seasonally hypoxic basins (Seitaj et al., 2015; Sulu-Gambari et al., 2016), and carbonate sands (Yin et al., 2021), as well as freshwater environments, such as lake sediments (Sachs et al., 2022), streambeds (Risgaard-Petersen et al., 2015; Liu et al., 2021), and groundwater systems (Müller et al., 2015; Müller et al., 2020). Within these environments, they strongly affect the biogeochemical transformations and fluxes in the sediment, e.g., by acidifying deeper sediment layers, which increases the dissolution of minerals and metals (Risgaard-Petersen et al., 2012; Malkin and Meysman, 2015; Seitaj et al., 2015; Rao et al., 2016; Sulu-Gambari et al., 2016; Van de Velde et al., 2016; Van de Velde et al., 2017).

This impact on sediment biogeochemistry is mediated by the cable bacteria’s capacity to generate and guide electrical currents over macroscale distances (Nielsen et al., 2010; Pfeffer et al., 2012; Malkin et al., 2014; Nielsen and Risgaard-Petersen, 2015; Bjerg et al., 2018; Meysman, 2018). These sulfur-oxidizing bacteria possess a unique “electrogenic” metabolism, where electrons from the anodic end of a filament in the sulfidic zone are transported all the way to the cathodic end in the oxic zone (Meysman et al., 2019). This way, electrons originating from sulfide oxidation can be internally transported from deeper sediment horizons toward oxygen near the sediment-water interface. This long distance electron transport (LDET) is mediated by an elaborate internal conductive structure, which consists of a set of nickel-containing protein fibers embedded in the periplasm as well as a conspicuous cartwheel structure in the cell–cell interfaces that connects these fibers (Cornelissen et al., 2018; Meysman et al., 2019; Boschker et al., 2021; Smets et al., 2024).

LDET appears to be a successful evolutionary adaptation, and occurs within a wide range of sediment environments (Meysman et al., 2019). This poses the question on the diversity of the actors that carry out LDET. Cable bacteria form a clade within the Desulfobulbaceae family of the Desulfobacterota phylum (Trojan et al., 2016), but their diversity has not been analyzed systematically. Up to present, nine species of the genus *Candidatus* Electrothrix and four species of the genus *Candidatus* Electronema are named and described (Trojan et al., 2016; Thorup et al., 2021; Geelhoed et al., 2023; Sereika et al., 2023; Hiralal et al., 2024a; Hiralal et al., 2024b; Plum-Jensen et al., 2024). Further, *Ca.* Electrothrix will be abbreviated to *Ca.* E. and *Ca.* Electronema to *Ca.* En. Additionally, two potential new genera, referred to by the code names AR3 and AR4, have been identified based on metagenome data, but not yet named and described (Geelhoed et al., 2023). However, it was proposed that the diversity of cable bacteria is largely underexplored (Marzocchi et al., 2014; Geelhoed et al., 2020), so that many more species and genera likely remain undiscovered. Until now, studies have mainly focused on fully marine or fully freshwater environments, while the diversity and physiology of cable bacteria in brackish environments such as estuaries have received less attention (Dam et al., 2021). Freshwater, brackish and marine environments typically display distinct microbiomes and there is evidence for a global brackish microbiome in water samples (Hugerth et al., 2015; Jurdzinski et al., 2023), and so the expectation is that brackish sediments may harbor an unrecognized diversity of cable bacteria.

Likewise, several questions remain on the relation between the diversity and biogeography of cable bacteria. Multiple cable bacteria species are known to coexist in the same sediment environment (Marzocchi et al., 2018; Geelhoed et al., 2023), but there has not been a comprehensive analysis of the possible environmental drivers that separate different taxonomic groups of cable bacteria. Overall, the environmental niches occupied by cable bacteria and the drivers of their diversity remain poorly understood (Burdorf et al., 2017; Dam et al., 2021). While salinity is hypothesized to be a key driver of cable bacteria diversity, other parameters such as the availability of oxygen in the bottom water and the diffusive supply of H_2_S were suggested to increase cable bacteria abundances (Hermans et al., 2019). The supply of oxygen and sulfide may also impact competition with other sulfur-oxidizing microbes such as Beggiatoaceae (Seitaj et al., 2015; Lipsewers et al., 2017; Liau et al., 2022; Malkin et al., 2022). Sulfide is the key electron donor of cable bacteria, and its availability could influence the coexistence of cable bacteria species. A higher availability of sulfide in the environment may reduce inter-species competition among cable bacteria and thus allow less dominant species to grow (Xu et al., 2022). Water quality, contaminant stress and access to sulfide are also considered to affect the diversity of cable bacteria assemblages in river sediments (Dong et al., 2022).

The aim of this study was to refine our knowledge of cable bacteria diversity, particularly within brackish and marine sediment environments. To this end, we compiled a dataset of complete or nearly complete 16S rRNA gene sequences. This approach enables a higher taxonomic resolution than the short amplicon sequences of isolated (hyper)variable 16S regions that are traditionally used in microbial community analysis (Johnson et al., 2019). To arrive at a database of long, high-quality 16S rRNA sequences, we followed two procedures. Firstly, existing 16S rRNA sequences were collected from literature and public databases along with metadata of the respective sampling sites. Secondly, novel 16S rRNA gene sequences of cable bacteria were generated from targeted laboratory enrichments of natural sediments with different salinities. This dataset was then used to evaluate the phylogenetic diversity of cable bacteria, and investigate their habitats and geographical distribution.

## 2 Materials and methods

### 2.1 Sampling sites and sediment collection

Enrichment incubations were performed in the laboratory to arrive at novel strains of cable bacteria. To this end, natural sediment was collected at 10 sampling sites in six different geographical locations (Table 1). Both brackish and marine sites were investigated, in order to cover a wide range of salinities. The salinity (S) in the overlying water was recorded at the time of sampling. Marine sediments were collected from the Ebro delta in Spain (S = 33), the Rattekaai salt marsh in the Netherlands (S = 30) and three subtidal sites in the Belgian Coastal Zone (BCZ) of the North Sea (S = 32). Brackish sediments were obtained from three sites in the Magazzolo estuary in Sicily, Italy (S = 2, 3 and 20), one site from the Yarra River estuary in Melbourne, Australia (S = 17), and one site in the Ebro delta in Spain (S = 10). A more detailed site description is given in the Supplementary methods.

**Table 1:** Sampling locations of bulk sediment for cable bacteria enrichment incubations.

Site code	Location	Latitude N	Longitude E	Habitat	Salinity (S)	Water depth [m]
ME2	Magazzolo Estuary, Sicily, Italy	37.4276	13.2509	River bank in estuary. Fine, gray, non-sulfidic sediment	2	<0.5
ME3	Magazzolo Estuary, Sicily, Italy	37.4287	13.2506	River bank in estuary. Fine, light gray, non-sulfidic sediment	3	<0.5
ME20	Magazzolo Estuary, Sicily, Italy	37.4274	13.2492	Isolated lagoon next to estuary, dark gray color, non-sulfidic	20	<0.5
ED10	Ebro Delta, Spain	40.6364	0.7473	Intertidal salt marsh. Sediment with interspersed black and brown layers	10	<0.5
ED33	Ebro Delta, Spain	40.6401	0.7399	Intertidal salt marsh. Dark-gray colored, sandy sediment	33	<0.5
BCZ130	Belgian Coastal Zone, North Sea	51.2688	2.9032	Subtidal marine sediment. Cohesive clay and mud. No bioturbation	30	10
BCZ700	Belgian Coastal Zone, North Sea	51.3687	3.2210	Subtidal marine sediment. Cohesive gray mud. Brittle stars	30	10
BCZOH	Belgian Coastal Zone, Ostend harbor, North Sea	51.2333	2.9261	Subtidal marine harbor sediment. Sulfidic black mud. No bioturbation	34	13
RSM30	Rattekaai Salt Marsh, The Netherlands	51.2621	4.1011	Intertidal creek bed of salt marsh. Black, sulfidic and fine-grained sediment. No bioturbation	30	<0.5
YR17	Yarra River, Melbourne, Australia	−37.8338	145.0258	Periodically hypoxic estuary site, high seasonal salinity fluctuations. Fine-grained, black, sulfidic sediment	17	2

Sediments from the deeper sites within the North Sea were collected with a Van Veen grab, which recovered the top ∼30 cm layer of the sediment. All the other sampling sites had shallow water (<0.5 m water depth), and here, the top 5–10 cm of sediment was collected manually with a plastic shovel. Sediments were stored in closed plastic containers with an overlying water layer, in the dark for a maximum of 8 weeks at room temperature (18–20°C) until the laboratory incubations were initiated.

### 2.2 Laboratory enrichment incubations and filament collection

Collected sediments were sieved (1.4 mm mesh size) and homogenized, and then packed into plexiglass core liners (4 cm inner diameter, 10 cm height) as described in Malkin et al. (2014). Three or more replicate sediment cores were incubated per site in the dark at room temperature. Each set of cores was placed in a separate non-transparent plastic box and submerged in artificial seawater (Instant Ocean® sea salt). The salinity of the artificial seawater was made to match the *in situ* salinity recorded (Table 1). The water was continuously aerated by bubbling with an aquarium pump.

The metabolic activity of the cable bacteria in the sediment cores was monitored through time. Cable bacteria induce a typical geochemical fingerprint in the sediment, which constitutes a suboxic zone in which neither O_2_ nor H_2_S are detectable (concentration <1μM) and a distinct pH profile that shows a subsurface pH maximum in the oxic zone and a pH minimum in the anoxic zone (Nielsen et al., 2010; Meysman et al., 2015). The development of cable bacteria activity was monitored by recording O_2_, H_2_S and pH depth profiles with microsensors as described before (Nielsen et al., 2010; Malkin et al., 2014). Once the characteristic geochemical fingerprint was identified in the incubation cores, individual filaments of cable bacteria were retrieved manually. Under a stereomicroscope, single filaments were extracted from the sediment with custom-made glass hooks and washed in drops of Milli-Q water to remove sediment particles (Pfeffer et al., 2012; Burdorf et al., 2017; Li et al., 2022). Cleaned filaments were then individually transferred into sterile PCR tubes (0.2 mL volume, VWR, United States) and stored at −20°C until used for PCR and sequencing. Filaments of species *Ca.* Electronema aureum GS and *Ca.* Electrothrix antwerpensis GW3-4 were manually picked from existing clonal cultures (Thorup et al., 2021; Hiralal et al., 2024b) and used exclusively for AFM imaging.

### 2.3 Microscopic imaging

Individual filaments were imaged using Atomic Force Microscopy (AFM) in order to investigate the morphology of the outer surface (i.e., to verify the presence of parallel ridges that harbor the periplasmic fibers that enable LDET in cable bacteria). To this end, cleaned cable bacteria filaments were transferred to a droplet of Milli-Q on a round glass coverslip and dried at room temperature. Coverslips were then mounted to magnetic metal disks of 20 mm diameter using double-sided carbon stickers. Images were recorded on an XE-100 AFM system (Park Systems) operating in tapping mode, using an aluminum SPM probe with a tip radius of <10 nm (AppNano ACTA-200) and with a nominal spring constant of 13–77 N/m. Topography and amplitude images were recorded and processed with the software Gwyddion (Nečas and Klapetek, 2012). To link morphology and phylogeny, filaments retrieved from the same sediment were used for both AFM imaging as well as for full-length 16S rRNA gene sequencing (see section 2.4). Note however that AFM and DNA sequencing were still applied to different filaments, and sometimes, DNA sequences from different strains were retrieved from the same sediment. Therefore, a given AFM morphology was only unequivocally assigned to a strain, when all 16S rRNA gene sequences retrieved from the particular sediment were identical.

### 2.4 16S rRNA gene sequencing of single filaments

Full-length 16S rRNA gene sequences were obtained from individual filaments by implementing a nested PCR protocol. Initial cell lysis was achieved by adding 15 μL of PCR water to the PCR tube containing the isolated filament and heating the sample at 95°C for 5 min. The first PCR reaction (PCR1) was executed to amplify the complete 16S rRNA gene using universal primers 27F and 1492R (Table 2). Each PCR tube contained a total volume of 20 μL: 15 μL of lysed cells and a master mix composed of 1.5 μL PCR water, 2 μL of DreamTaq Buffer (10X), and final concentrations of 0.2 mM dNTPs, 0.5 μM forward primer, 0.5 μM reverse primer and 0.025 U/μL DreamTaq DNA Polymerase (Thermo Scientific, United States). The cycling conditions for PCR1 were: initial denaturation at 95°C for 2 min, 30 cycles of denaturation at 95°C for 45 s, annealing at 46°C for 45 s, extension at 72°C for 1:30 min and final extension at 72°C for 10 min.

**Table 2:** Primers used to amplify the 16S rRNA gene of single cable bacteria filaments.

Name	Primer sequence (5′ – 3′)	Size [nt]	References
27F	AGA GTT TGA TCM TGG CTC AG	20	Giovannoni (1991)
39Fc^a^	GGC TCA GAA CGA ACG CTG	18	Modified after Hongoh et al. (2003) and Geelhoed et al. (2023)
64Fc^b^	RTG CTT AAC ACA TGC AAG TCG	21	Modified after Hongoh et al. (2003)
1492R	GGY TAC CTT GTT ACG ACT T	19	Loy et al. (2005)
DSBB280wF	CGA TGG TTA RCG GGT CTG	18	Geelhoed et al. (2020)
DSBB+1297R	AGA CTC CAA TCC GGA CTG A	19	Geelhoed et al. (2020)

a Primer 39Fc is 1 base shorter, at the 5′ end, and lacks the degenerate bases compared to primer 39F (Hongoh et al., 2003).

b Primer 64Fc is 1 base longer, at the 5′ end, with a T instead of a C at position 5, and lacks the degenerate bases compared to primer 64F (Hongoh et al., 2003).

For the second PCR reaction (PCR2) combinations of universal primers (27F, 1492R) and Desulfobulbaceae-specific primers (DSBB280wF, DSBB+1297R) were used: 27F-DSBB+1297R, and DSBB280wF-1492R (Table 2; Supplementary Figure S1). Because the amplification with primer pair 27F-DSBB+1297R was only successful in 42% of amplified PCR1 products, the alternative pairs 39F-DSBB+1297R and 64F-DSBB+1297R were tested to improve amplification success and sequence length. Each reaction contained a total volume of 20 μL prepared as mentioned for PCR1. PCR2 used 2 μL of tenfold dilutions of PCR1 products and cycling conditions were the same for all primer sets, differing to PCR1 cycling conditions in that annealing was performed at 50°C for 45 s and the extension was shortened to 1 min at 72°C (Table 2). Amplification of PCR2 products was confirmed via 1% agarose gel electrophoresis.

The PCR2 amplicon products were Sanger sequenced (Center for Molecular Neurology, VIB-UAntwerpen, Belgium) and the resulting sequences were manually checked and curated. A multiple sequence alignment of the individual partial sequences was conducted using MUSCLE (Edgar, 2004) before assembling them to consensus sequences of the 16S rRNA gene with lengths varying from *ca.* 800 to 1,500 bp.

### 2.5 16S phylogenetic tree construction and taxonomic clustering

The available high-quality (i.e., sufficiently long, ≥800 bp) 16S rRNA gene sequences in the dataset were grouped into “species-level clades” and “genus-level clusters.” Note that this clustering approach is exclusively based on DNA relatedness, i.e., similarity of sufficiently long 16S rRNA gene sequences. While it allows a good indication of the genus-level and species-level diversity, it is not sufficient to effectively name and describe taxa, as the taxonomic delineation of species and genera requires additional biochemical and other phenotypic data (Rosselló-Móra and Amann, 2015).

Our clustering approach consisted of three separate steps. In a first step, pairwise gene sequence identities were computed with the distance matrix tool in MEGA (version 10.2.5). To define a species-level clade, we applied the conventional 16S rRNA gene sequence identity cutoff of 98.7% (Yarza et al., 2014). The groupings thus obtained were recognized as genuine “species-level clades” when one additional criterium was fulfilled: the cluster should contain at least one nearly complete 16S rRNA gene sequence (sequence of ≥1,200 bp), as to increase the reliability of the taxonomic assignment.

In a second step, we constructed a phylogenetic tree, using the longest sequence of each species-level clade. This selection process was crucial for achieving a reliable phylogenetic tree, as large numbers of sequences can lead to more frequent multifurcations (Boyce et al., 2015). Initially, all cable bacterium sequences were aligned along with reference sequences of closely related Desulfobulbales species (Supplementary Table S1) using the MUSCLE algorithm (Edgar, 2004). The phylogenetic tree was calculated in IQ-TREE v1.6.12 with the automatic best-fit model finder and 1,000 ultrafast bootstrap iterations (Nguyen et al., 2015) using the 16S rRNA gene sequence of *Geobacter sulfurreducens* PCA (NR_075009) as outgroup. The tree was visualized in FigTree (version 1.4.4).

In a third step, we calculated pairwise gene sequence identities for the 90 reference sequences selected in the species-level clades. The conventional genus-level cutoff value of 94.5% sequence identity (Yarza et al., 2014) was used in combination with the tree topology and grouping of species-level clades to delineate genus-level clusters.

## 3 Results

### 3.1 Long 16S rRNA gene sequences from single filaments

To achieve a reliable taxonomic resolution at the species level, long to full-length 16S rRNA gene sequences (800–1,500 bp) are required (Kim et al., 2011). Such sequences were obtained by targeted incubation of sediments from 10 different marine and brackish locations, followed by retrieval of individual filaments from the sediment, nested PCR application and subsequent Sanger sequencing. The first PCR reaction (PCR1) was always executed using universal primers 27F and 1492R (Table 2). For the second PCR reaction (PCR2), we initially used the primer set DSBB280wF-DSBB+1279R, generating 16S sequences of *ca.* 900 to 1,000 bp. To attain longer sequences, we adjusted the primer pair (27F/39Fc/64Fc-DSBB+1297R and DSBB280wF-1492R; Table 2), which provided sequences within the range from 1,464 to 1,515 bp (average length: 1,495 bp). In this manner, 125 sequences from single cable bacterium filaments were obtained with ≥800 bp, 26 of which were full-length 16S rRNA gene sequences (≥1,450 bp, Table 3). Overall, 86 sequences were retrieved from marine sediments and 39 sequences were obtained from brackish sediments.

**Table 3:** Sources and numbers of cable bacteria (CB) 16S sequences before and after quality control and filtering.

Source	Initial # sequences	# potential CB sequences ≥ 92%	# within monophyletic CB clade^a^	# long 16S sequences ≥ 800 bp	Unique clade (≥98.7%)
Laboratory enrichment	125	125	125	125	6
Literature compilation^b^	206	206	163	119	28
SILVA	5,469	1,802	1,615	1,547	21
NCBI GenBank	204	204	85	85	35
Total	6,004	2,337	1,988	1,876	90

a In this step, poorly aligned sequences were also removed.

b Extracted from genomes of Thorup et al. (2021), Geelhoed et al. (2023), Sereika et al. (2023), Hiralal et al. (2024a), Hiralal et al. (2024b), Plum-Jensen et al. (2024) and 16S sequences provided by Sachs et al. (2022).

### 3.2 Sequence data mining

In addition to the novel 16S rRNA gene sequences obtained from single cable bacteria filaments, existing cable bacteria sequences were compiled from the literature and public databases. A total of 206 sequences were collected from literature sources, including the pioneering study by Trojan et al. (2016) and other cable bacteria studies (Pfeffer et al., 2012; Malkin et al., 2014; Marzocchi et al., 2014; Schauer et al., 2014; Larsen et al., 2015; Xu et al., 2021; Yang et al., 2023) as well as from recently published cable bacteria genomes by Thorup et al. (2021), Geelhoed et al. (2023), Sereika et al. (2023), Hiralal et al. (2024a), Hiralal et al. (2024b), and Plum-Jensen et al. (2024). The sequences of ribosomal RNA genes in the available genomes were retrieved using barrnap v0.9.^1^ Additionally, 49 full-length 16S sequences were generated through PacBio sequencing as documented in Sachs et al. (2022) (Table 3).

Cable bacteria sequences were additionally compiled from two public databases: the SILVA rRNA database and NCBI GenBank. The SILVA 138.1 small subunit rRNA database (release SSU Parc, accession date: 7 July 2024) is a public repository for 16S rRNA sequence datasets (Quast et al., 2012) which implements automatic taxonomic classification of the sequences. To obtain potential cable bacteria sequences, we extracted 1,630 sequences assigned to the genera *Ca.* Electrothrix and *Ca.* Electronema (excluding the 71 sequences that were already extracted from literature) as well as 3,839 unassigned Desulfobulbaceae sequences (Table 3). To determine whether 16S sequences were truly belonging to the cable bacteria clade, stand-alone blastn (Altschul et al., 1990; Camacho et al., 2009) was used to screen these sequences against a reference set of known cable bacteria sequences. This reference query set contained five sequences that covered the spectrum of the currently known diversity of cable bacteria: *Ca.* Electrothrix communis (KR912339), *Ca.* Electronema palustre (KP728463, Trojan et al., 2016), *Ca.* Electronema halotolerans (GCA_942493095, Sereika et al., 2023) and the potential genera AR3 (GCA_022765765, Geelhoed et al., 2023) and AR4 (GCA_022765725, Geelhoed et al., 2023). Note that this approach inherently bears an important constraint: we specifically screen the diversity within the main monophyletic *Ca.* Electrothrix/*Ca.* Electronema clade. Therefore, cable bacteria sequences that fall outside this clade—if such would exist—will not be retained by the approach.

NCBI GenBank (Release 255, accession date: 7 July 2024) is a genetic sequence repository which contains the nucleotide collection (nr/nt) database with publicly available 16S nucleotide sequences (Benson et al., 2018). Unlike the SILVA database, NCBI GenBank does not automatically classify sequences taxonomically. Thus, to identify potential cable bacteria 16S sequences, blastn was used to screen the default nucleotide collection (nr/nt) database of GenBank against the same reference query set as used above. A sequence identity of ≥92% between any of the five query cable bacteria sequences and the sequences in the database was used as selection criterion to extract potential cable bacteria 16S rRNA gene sequences. This way, 204 additional sequences were identified that were not included in the sequences extracted from the SILVA database nor compiled from literature (Table 3). When available, corresponding metadata was compiled from each sequence based on the information provided in the databases including the habitat type, salinity, and sulfide concentration. Sediments were classified into four categories based on their salinities: freshwater (S < 0.5), brackish (0.5 ≤ S < 30), marine (30 ≤ S ≤ 36) and hypersaline (S > 36).

### 3.3 Curation of compiled 16S rRNA gene sequences

The compiled database of 16S rRNA sequences was curated based on two criteria. In a first step, the 16S sequence identity was screened: only sequences that had an identity value ≥92% compared to any of the five cable bacteria query sequences (as defined in section 3.2) were retained. Of the 5,469 extracted SILVA sequences, 1802 (33%) met this 92% cutoff criterion (Table 3). This included all sequences previously assigned to *Ca.* Electrothrix or *Ca.* Electronema in literature, but with one exception. Sequence GU208270 was retained although it only showed a maximum identity of 90.9% to the query set. However, it is classified as cable bacterium *Ca.* Electronema in the SILVA database and has been recognized as a putative cable bacterium sequence in previous studies (Trojan et al., 2016; Scholz et al., 2021). Sequences collected from NCBI GenBank already fulfilled the sequence identity ≥92% criterion, so these were not filtered any further. Likewise, all sequences obtained from literature and single filaments fulfilled the sequence identity cutoff criterion. As a result, 2,337 sequences were retained in total as potential cable bacteria sequences (Table 3).

As a second criterion, the sequences had to phylogenetically cluster within the main monophyletic *Ca.* Electrothrix/*Ca.* Electronema clade to confirm their taxonomic affiliation as cable bacteria. To this end, the 2,337 retained sequences were aligned with closely related Desulfobulbales taxa (Supplementary Table S1) using MUSCLE (Edgar, 2004). After manually examining the alignment, 71 sequences that contained indels and mutations in conserved regions of the 16S rRNA gene were removed from the dataset. The phylogenetic placement of the aligned sequences was evaluated by constructing a phylogenetic tree in IQ-TREE. A total of 278 sequences were additionally removed as they clustered outside of the cable bacteria monophyletic clade. For the remaining 1,988 cable bacteria sequences (Supplementary Table S2) a sequence length cutoff of 800 bp was applied to ensure sufficient, species-level taxonomic resolution (White et al., 2010). A total of 112 sequences were shorter than 800 bp and were excluded from the phylogenetic analysis (Table 3). The final dataset used for phylogenetic analysis hence included 1,876 sequences in total (Table 3).

### 3.4 Phylogenetic tree of cable bacteria

**Figure 1:**
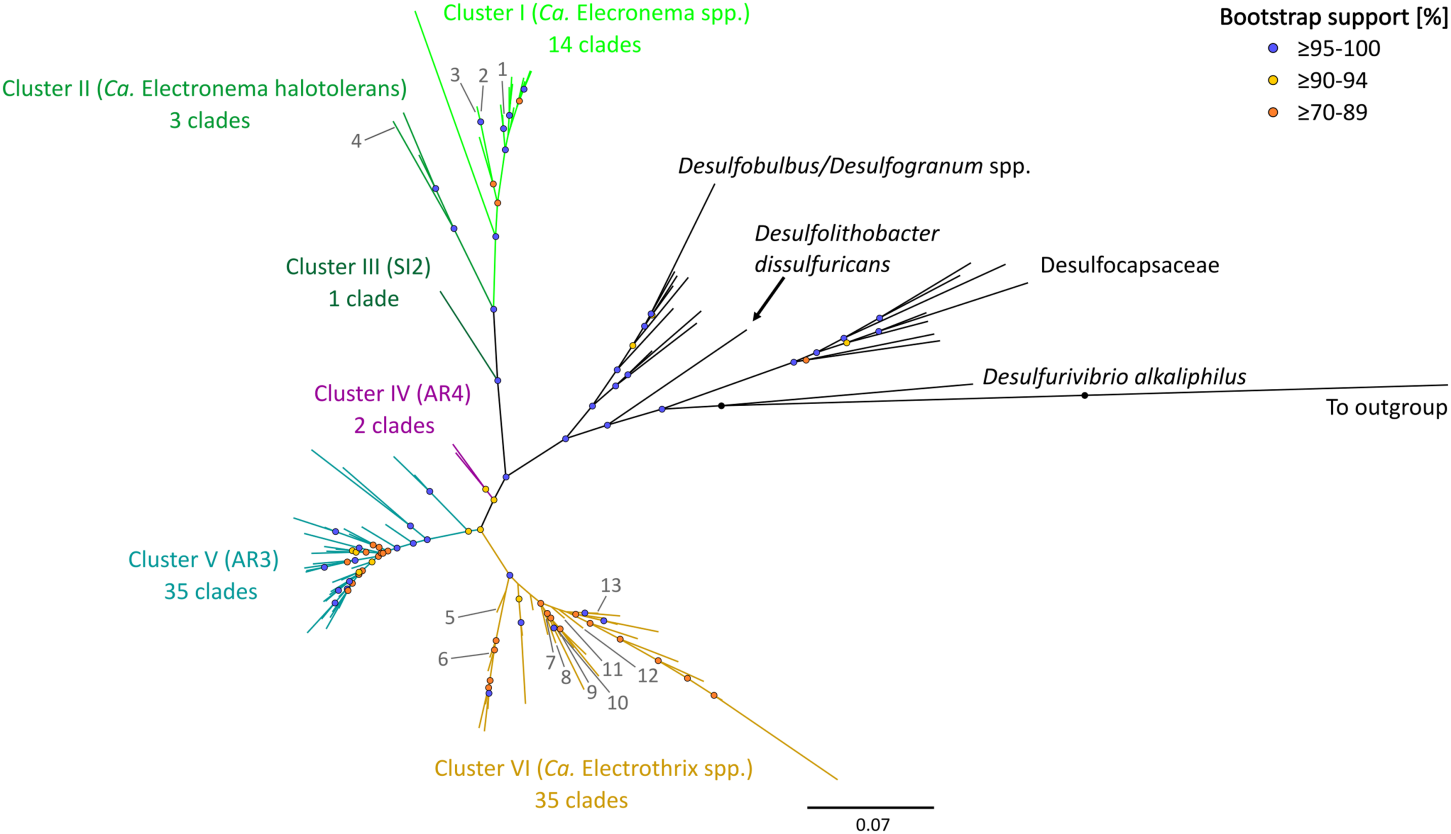
16S rRNA gene phylogenetic tree of the six clusters containing 90 cable bacteria species-level clades. Colored nodes indicate bootstrap support (1,000 iterations) and colored branches indicate the different clusters. Phylogeny was inferred using IQ-TREE according to the best-fit model TIM3e + I + G4. Previously described cable bacteria species are annotated with numbers 1–13: (1) *Ca.* Electronema palustre, (2) *Ca.* Electronema nielsenii, (3) *Ca.* Electronema aureum, (4) *Ca.* Electronema halotolerans, (5) *Ca.* Electrothrix marina, (6) *Ca.* Electrothrix aestuarii/scaldis, (7) *Ca.* Electrothrix communis, (8) *Ca.* Electrothrix rattekaaiensis, (9) *Ca.* Electrothrix laxa, (10) *Ca.* Electrothrix aarhusiensis, (11) *Ca.* Electrothrix japonica, (12) *Ca.* Electrothrix antwerpensis, (13) *Ca.* Electrothrix gigas. All sequences used are listed in Supplementary Table S4. The same tree in rectangular view with tip labels is shown in Supplementary Figure S3.

The updated phylogenetic tree of cable bacteria contains six distinct genus-level clusters and 90 species-level clades, all within a monophyletic clade forming a sister group to the *Desulfobulbus* and *Desulfogranum* genera within the Desulfobulbaceae (Figure 1; Table 3).

Pairwise sequence similarity analysis, using the sequence identity cutoff of 98.7% (Yarza et al., 2014), clustered the 1,876 available 16S rRNA gene sequences in the dataset into 98 separate groups. Among these, 90 groups fulfilled the additional criterion of containing at least one sequence that was sufficiently long (≥1,200 bp), and hence, these groups were designated as genuine species-level clades (85 clusters contained at least one 16S rRNA gene sequence longer than 1,400 bp, while for the other 5, the longest available sequence ranged between 1,200 and 1,400 bp). Note that 38 species-level clades (42%) only contained a single sequence, while the other species-level clades were represented by multiple sequences (up to 1,083 sequences; Supplementary Table S3; Supplementary Figure S2).

**Figure 2:**
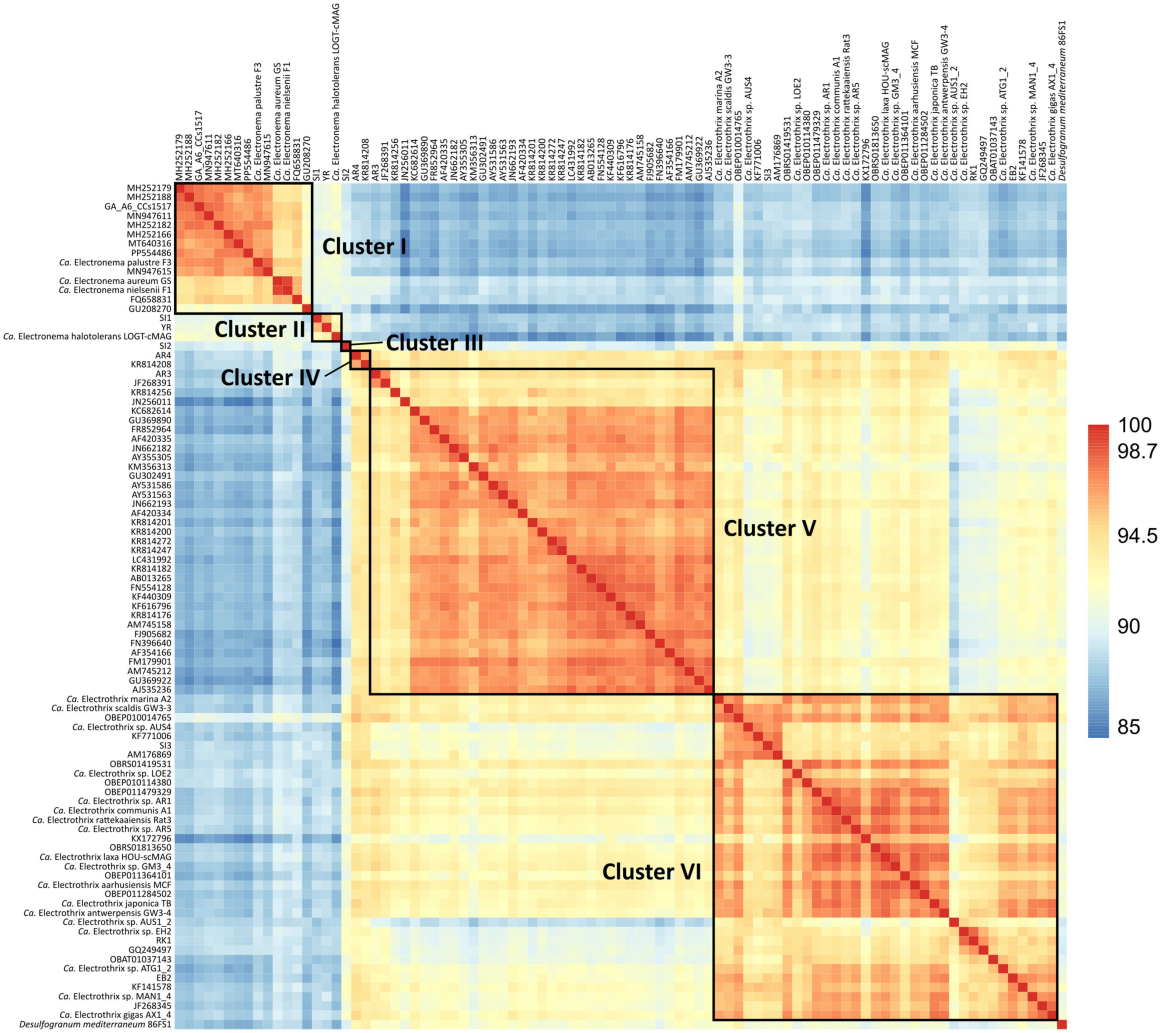
Heatmap of 16S rRNA sequence identities of the 90 cable bacteria species-level clades and their closest relative *Desulfogranum mediterraneum*. Colors indicate % sequence identity.

The 90 species-level clades were used to construct the 16S based phylogenetic tree (Figure 1), as well as a heatmap of 16S rRNA sequence identity (Figure 2). Combining these two types of information, 6 genus-level clusters were delineated. The genus-level cutoff of 94.5% identity was used as a guide value to define the clusters (Figure 2) but final clusters were mainly determined based on the phylogenetic clustering of species-level clades (Figure 1). Therefore, the genus-level clustering does not always follow the 94.5% identity cut-off that has been traditionally proposed to delineate genera based on 16S rRNA gene sequences (Yarza et al., 2014). Comparing sequence identities among the six genus-level clusters, the minimum sequence identity within one cluster (intra-cluster sequence identity) was 89.7% (AUS1_2 vs. KX172796), while the maximum sequence identity between clusters (inter-cluster sequence identity) was 95.9% (AR4 vs. OBEP010014765). Likewise, the species diversity greatly varied among the 6 clusters, and ranged from only one species-level clade (Cluster III) up to 35 species-level clades (Clusters V and VI) per genus-level cluster (Table 4).

**Table 4:** Sequence information and environmental parameters of the six cable bacteria clusters.

Parameters/clusters	I	II	III	IV	V	VI
# sequences	125	26	5	17	78	1,625
Genus name/code	*Ca.* Electronema	*Ca.* Electronema	SI2	AR4	AR3	*Ca.* Electrothrix
# species-level clades (≥1,200 bp)	14	3	1	2	35	35
# species-level clades (<1,200 bp)	2	0	0	0	0	6
New proposed species	0	SI1, YR	SI2	0	0	SI3, EB2, RK1
# sites	11	5	1	3	23	35
Salinity	<0.5	2–20	3	30–32	32	0–50
Type	Freshwater	Brackish	Brackish	Marine	Marine	Freshwater to hypersaline
Free sulfide availability	n.a.	low	low	low	low–high	low–high
Water depth [m]	0.3–0.5	0.3–2.0	0.3	10–988	10–3,038	0–3,297

Free sulfide concentrations classification: low: 0–10 μM, medium: 10–500 μM, high: >500 μM.

In the following sections we provide an overview of the six different genus-level clusters and the species-level clades they contain. Previously described taxa are referred to by their name, while as yet undescribed species-level clades are given a short code name. Note that recently a new cable bacteria taxonomy was proposed following the rules of the Code of Nomenclature of Prokaryotes Described from Sequence Data (SeqCode), thus removing the *Candidatus* (*Ca.*) designation for those cable bacteria for which high quality genomes are available (Hedlund et al., 2022; Plum-Jensen et al., 2024). Yet, because of lacking genome data, not all of the named cable bacteria species could be validated under the SeqCode. Hence for consistency, we will use the original species names and keep the *Candidatus* (*Ca.*) designation for all previously named species.

#### 3.4.1 Cluster I

**Figure 3:**
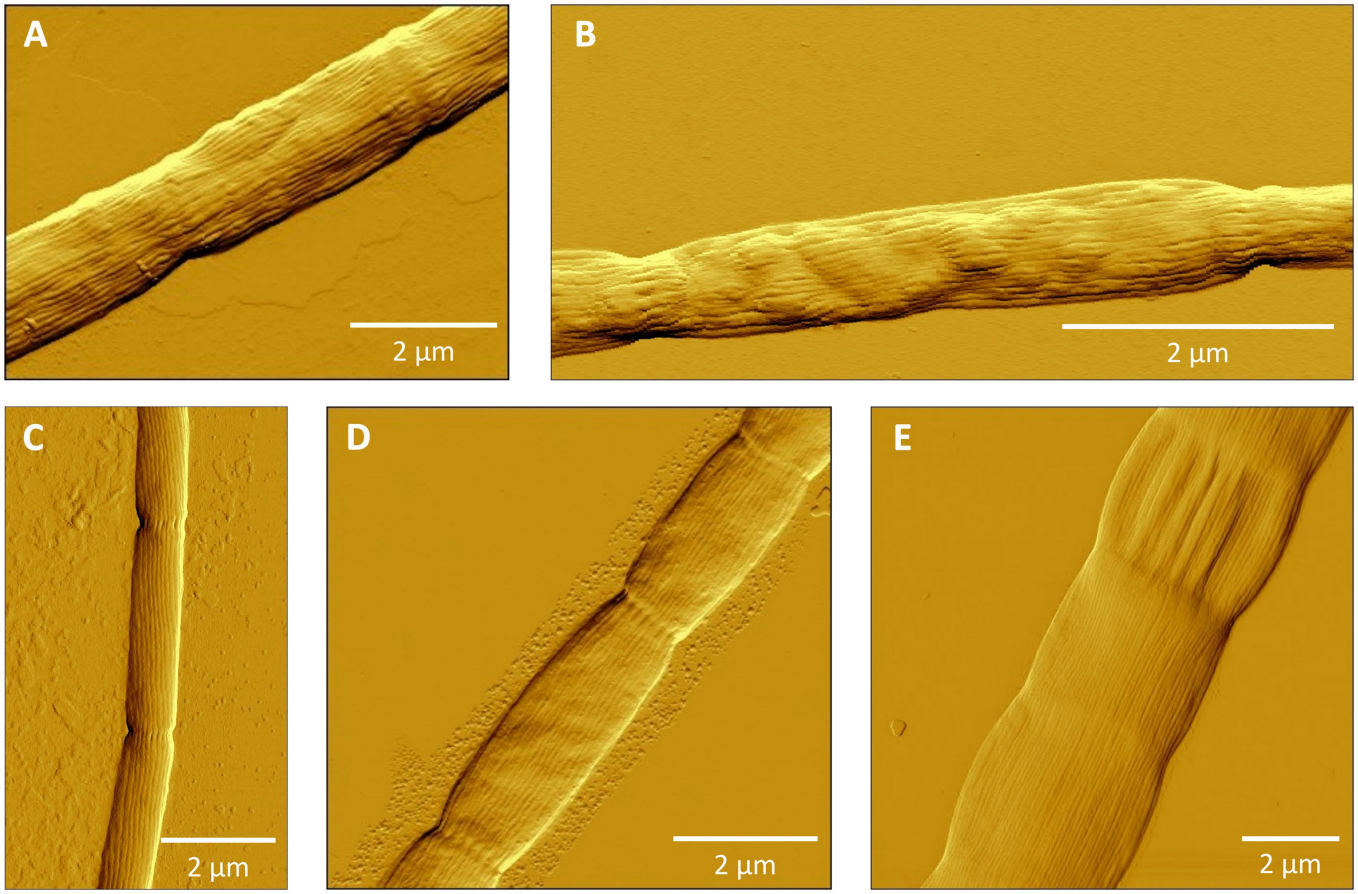
Atomic force microscopy (AFM) images of filamentous cable bacteria. **(A)** Cluster I: *Ca.* Electronema aureum GS, **(B)** Cluster II (new species SI1) or Cluster III (new species SI2), **(C)** Cluster VI: *Ca.* Electrothrix antwerpensis GW3-4, **(D)** Cluster VI: new species EB2, **(E)** Cluster VI: *Ca.* Electrothrix gigas. No images of Clusters IV and V were available.

Cluster I contains 14 species-level clades and corresponds to the originally described genus *Ca.* Electronema (Trojan et al., 2016), which is considered to have specific adaptions for freshwater environments (Sereika et al., 2023). Indeed, all sequences within Cluster I (*n* = 125) originate from genuine freshwater environments (salinity <0.5; Figure 1; Table 4), and our sediment incubations from marine and brackish sediments did not contribute any sequences to this cluster. Cluster I includes three previously described species *Ca.* En. palustre, *Ca.* En. nielsenii and *Ca.* En. aureum (Trojan et al., 2016; Thorup et al., 2021) as well as 11 new species-level clades. Sequences of *Ca.* En. palustre, nielsenii and aureum were obtained from a streambed and a freshwater artificial pond in Denmark (Trojan et al., 2016; Thorup et al., 2021) as well as river sediments in southern Germany and eastern China (Xu et al., 2021; Sachs et al., 2022). The 11 new species-level clades also originated from freshwater systems, including river sediments in China (MH252151–MH252199 and MT640316–MT640327) and Germany (Sachs et al., 2022), paddy soils (PP554486), as well as sediments from a freshwater pond in a restored wetland and a lake in South California (Yang et al., 2023). Reported filament diameters in clades within this cluster ranged from 0.7 to 3.0 μm (Figure 3A) (Trojan et al., 2016; Thorup et al., 2021; Yang et al., 2023).

#### 3.4.2 Cluster II

Cluster II contains three species-level clades and the associated sequences (*n* = 26) all originate from brackish systems with a salinity ranging from 2 to 23 (Figure 1; Table 4). The first species-level clade is represented by a single sequence of the recently described *Ca.* En. halotolerans, which has a filament diameter of 1 to 2 μm and was originally found at Loegten strand (a coastal beach in the Baltic Sea, Denmark) at an *in situ* salinity of *ca.* 18–23 (Sereika et al., 2023).

The second species-level clade (code name “SI1”) contains 14 sequences obtained from sediment incubations of three different sites within the Magazzolo river estuary in Sicily (ME2, ME3, ME20; Table 1). These sites were located within a 100 m distance of each other, but their salinities varied from 2 to 20 at the time of sampling, thus indicating a steep salinity gradient within this small estuary. Due to the tidal and storm surge fluctuations and variable freshwater discharge, high salinity fluctuations are expected at these sites. The incubated sediment of all three sites showed a low free sulfide concentration (<10 μM).

The third species-level clade (code name “YR”), with a diameter of *ca.* 1.0 μm, incorporates 11 sequences obtained from our sediment incubation from a brackish site (S = 17) within the Yarra River estuary (YR17; Table 1). This site, near Scotch College, has a highly variable salinity (5 ≤ S ≤ 30) and shows high free sulfide concentrations *in situ* (Burdorf et al., 2017; Kessler et al., 2019; Burdorf et al., 2024), although the sulfide concentrations in our sediment incubations were lower. Species-level clades SI1 and YR show a rather high sequence identity of 95.9% to each other, but a much lower identity to the recently described species *Ca.* En. halotolerans (92.5 and 93.5% identity, respectively) (Sereika et al., 2023). The latter values even fall below the conventional genus level cutoff of 94.5%. Still, we consider SI1, YR and *Ca.* En. halotolerans to belong to the same cluster, as the phylogenetic tree shows a distinct grouping (Figure 1).

#### 3.4.3 Cluster III

Cluster III contains only one species-level clade (code name “SI2”), which encompasses five sequences originating from our sediment incubations of the low-salinity sites ME2 and ME3 in the Magazzolo river estuary in Sicily (Table 1). The closest described relative of these sequences is *Ca.* E. scaldis GW3-3 at 92.2% sequence identity, while the closest relative within the Electronema genus is 90.6% identical (*Ca.* En. aureum). These sequence identities are far below the standard genus-level cutoff of 94.5%, while at the same time, the phylogenetic tree shows a distinct basal branching of Cluster III between the Clusters II and IV (Figure 1). Hence, Cluster III potentially represents a new cable bacteria genus. The sediment enrichments from which SI2 sequences were retrieved also provided filaments from Clusters II and VI, and so, the AFM images of picked filaments could not be unambiguously linked to their taxonomic identity. Thus we have no information on the morphological characteristics of the SI2 clade.

#### 3.4.4 Cluster IV

Cluster IV contains two species-level clades (Table 4). The cluster contains the AR4 species-level clade, which originated from the subtidal site BCZ130 in the North Sea (coastal zone of Belgium). An additional 14 sequences of AR4 were generated in the present study, of which 12 originated from site BCZ130 and two from the nearby BCZ700 site. Filament diameters ranged from 0.8 to 1.0 μm. Shorter 16S rRNA gene sequences (∼400 bp) assigned to AR4 have been reported from oxic basins in the Baltic Sea (7 ≤ S ≤ 21) (Dam et al., 2021) and sulfidic sediments underneath fish farms in Iceland (S > 30) (Vasquez-Cardenas et al., 2022). The closest described relative to AR4 is *Ca.* E. marina at 95.2% sequence identity. The second species-level clade within Cluster IV is formed by two sequences, which originate from sediments at a deep sea mud volcano in Costa Rica (KR814208) and a hydrocarbon seep in the Gulf of Mexico (GU302481) (Lloyd et al., 2010) (Figure 1). There is no morphological information on this clade.

#### 3.4.5 Cluster V

Cluster V shows a high diversity and contains 35 species-level clades of which the associated sequences all originate from marine environments. Cluster V includes the species-level clade AR3, which is represented by one full-length 16S sequence obtained from site BCZ130. At 95.0% sequence identity, *Ca.* E. communis is the closest described relative to AR3. At 97.2% sequence identity, sequence JF268391 from a deep sea sediment in New Zealand, belongs to the same genus. Additionally, 33 species-level clades were identified, of which 27 originate from deep sea sites (>700 m water depth). The majority of these sites were reduced environments such as hydrothermal vents and cold seeps (Supplementary Table S5). As such, Cluster V emerges as a deep sea clade of cable bacteria that has undergone a substantial radiation. Note that for a few of the species-level clades, the positioning within Cluster V remains uncertain, as the sequence identities to both AR3 (92.7–95.3%) and AR4 (92.6–95.1%) are similar and close to the genus-level cutoff (Figures 1, 2).

#### 3.4.6 Cluster VI

Cluster VI includes 35 species-level clades and corresponds to the previously described genus *Ca.* Electrothrix (Figure 1; Table 4). Of all the clusters, Cluster VI has received the most attention until present. Nine species are already described in literature: *Ca.* E. communis, aarhusiensis, marina, japonica (Trojan et al., 2016), laxa (Sereika et al., 2023), gigas (Geelhoed et al., 2023), rattekaaiensis (Plum-Jensen et al., 2024), antwerpensis (Hiralal et al., 2024b) and a species-level clade that is currently represented by two species that were recently proposed within 1 month from each other: *Ca.* E. aestuarii Rat1 (Plum-Jensen et al., 2024) and *Ca.* E. scaldis GW3-3 (Hiralal et al., 2024a). The first three species originate from nearshore sulfidic sediments in the Baltic Sea and were already described in the first taxonomic study devoted to cable bacteria (Trojan et al., 2016). *Ca.* E. communis and aarhusiensis occur in brackish and marine sediments (including salt marshes) and are able to tolerate a wide range of salinities (*Ca.* E. communis: 0.3–21, *Ca.* E. aarhusiensis: 0.3–35) (Trojan et al., 2016; Dam et al., 2021). However, one sequence of *Ca.* E. aarhusiensis was also detected in a freshwater lake (Karst et al., 2018). Recently, a diameter of ∼0.8 μm was reported for the strain *Ca.* E. communis RB (Plum-Jensen et al., 2024). *Ca.* E. marina was originally detected in Aarhus Bay sediment incubations at salinity 18 and our database search found three additional, almost identical sequences (99.9% identity) in Danish fjord sediments of unknown salinity (Karst et al., 2018). *Ca.* E. japonica was described from subtidal sediments within Tokyo Bay (Japan) (Trojan et al., 2016). *Ca.* E. laxa was found in sediment from Hou, which is also located in Aarhus Bay (Sereika et al., 2023) and at a shore in Northern Denmark (Karst et al., 2018). The clade of *Ca.* E. gigas contains 44 sequences, which were found in intertidal, highly sulfidic salt marsh sediments at different locations in the Netherlands (S ∼ 30), a brackish sediment in Denmark (18 ≤ S ≤ 23) and an estuary with high salinity fluctuations (5 ≤ S ≤ 30) and low free sulfide in Australia (Burdorf et al., 2017; Geelhoed et al., 2023; Sereika et al., 2023).

The final three species *Ca.* E. rattekaaiensis, antwerpensis, and aestuarii/scaldis (with strains Rat1 and GW3-3, respectively) were all retrieved from the same salt marsh site in the Netherlands, RSM30 (Hiralal et al., 2024a; Hiralal et al., 2024b; Plum-Jensen et al., 2024). This site has typically high levels of pore water sulfide, is rich in organic matter and has a fine grain size (Burdorf et al., 2017). Sequences belonging to *Ca.* E. aestuarii were also detected in a marine, non-sulfidic salt marsh near the Ebro delta (site ED33). An exploratory phylogenetic tree analysis of all short 16S rRNA gene sequences (<800 bp) compiled in this study (data not shown) identified another highly similar sequence (745 bp) in bioturbated sediment from the Gulf of Fos in the Mediterranean Sea (sequence identity: 99.6%) (Pischedda et al., 2011).

Additionally, our sediment incubations resulted in three novel species-level clades: SI3, EB2 and RK1 (Figure 1; Table 4). Clade SI3 contains two sequences from low-sulfide (<10 μM) incubations of site ME2 (S = 2) in Sicily where it was found to co-occur with species-level clade SI1 (Cluster II) and SI2 (Cluster III) (Figure 1). The closest described relative is *Ca.* E. scaldis GW3-3 at 96.3% sequence identity. Clade EB2 contains a total of eight sequences from the Ebro Delta, of which seven were recovered from a sulfidic salt marsh site ED10 at lower salinity (S = 10) and one from the close-by site ED33 at higher salinity (S = 33). At 97.5% sequence identity, *Ca.* E. antwerpensis is the closest described relative to this clade. Clades EB2 and SI3 are the first potential *Ca.* Electrothrix species reported from sediments with *in situ* salinities <15. Species-level clade RK1 contained 11 sequences, ten of which were obtained from the sediment incubations of the marine RSM30 site at salinity 30. Another sequence of RK1 was found in incubations of marine site BCZOH at salinity 34 (Table 1). The closest described relative of RK1 is *Ca.* E. gigas at 95.6% sequence identity.

Additionally, Cluster VI contains nine species-level clades (AR1, AR5, ATG1_2, AUS1_2, AUS4, EH2, GM3_4, LOE2, MAN1_4), which have been recently proposed based on partial genome data (43.5–86.5% estimated genome completeness), but are not fully described as species (Geelhoed et al., 2023). Lastly, Cluster VI includes 14 potential species-level clades that have been identified here based on 16S rRNA gene sequences extracted from SILVA and literature, of which eight originate from brackish or marine sediments in Denmark (Karst et al., 2018), as well as a previously identified cable bacterium from a salt marsh in New England (Larsen et al., 2015). Moreover, six potential species-level groups in this cluster did not contain any 16S sequences >1,200 bp, resulting in their exclusion from the phylogenetic tree.

**Figure 4:**
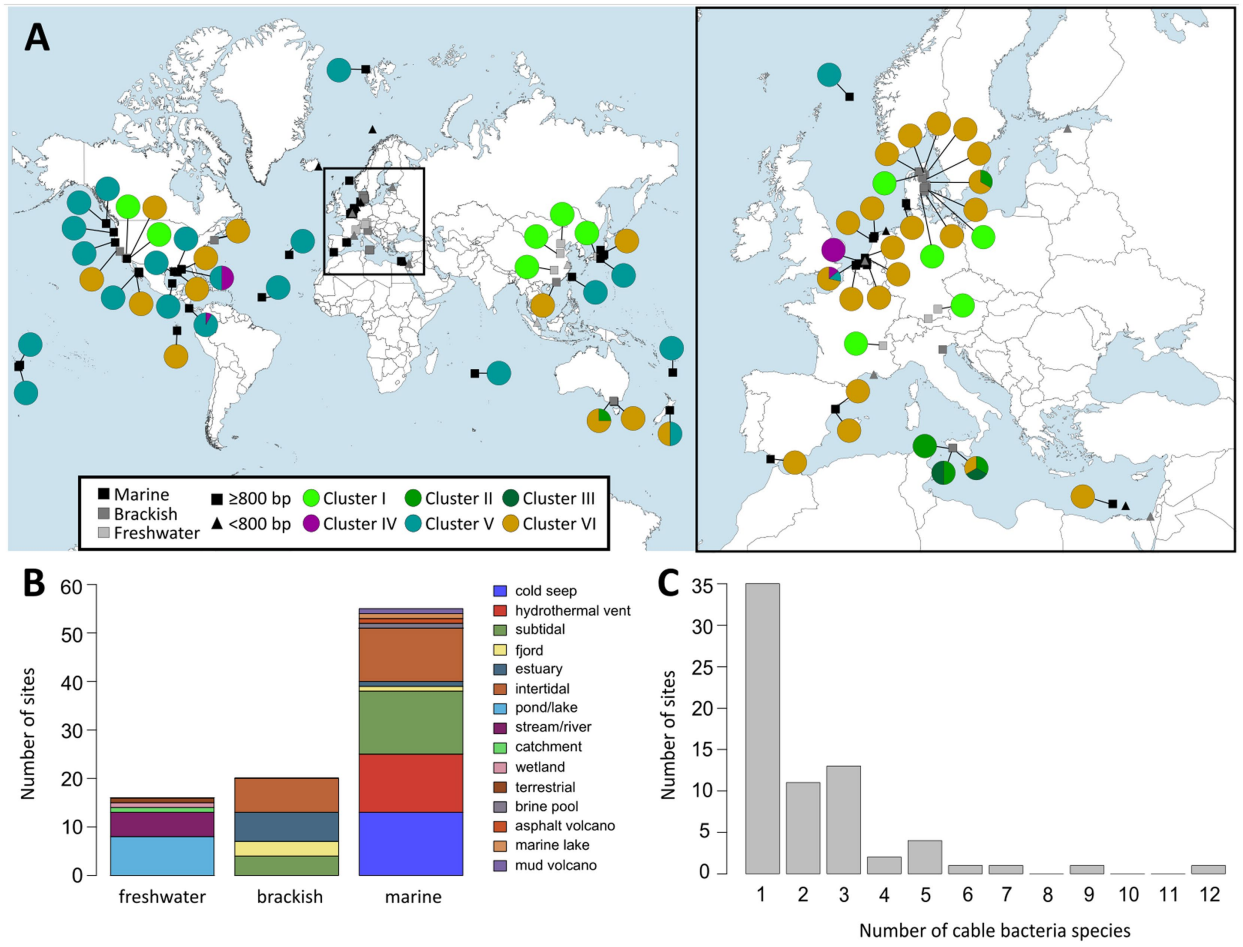
**(A)** World map (left) and map of Europe (right) showing locations at which cable bacteria 16S rRNA gene sequences were detected based on the two databases NCBI GenBank and SILVA 138. Rectangles indicate long (≥800 bp) and triangles indicate short sequences (<800 bp). Colored circles show pie charts indicating the proportion of cable bacteria from the different clusters found at each site. **(B)** Distribution of habitat types at which cable bacteria were found grouped by salinity category: freshwater (S < 0.5), brackish (0.5 ≤ S < 30) and marine (S ≥ 30). **(C)** Distribution of the number of different cable bacteria species-level clades present at the sampling sites.

Overall, all but one of the sequences of Cluster VI originated from brackish or marine salinities (2 ≤ S ≤ 36), thus supporting the broad salinity tolerance of the *Ca.* Electrothrix genus (Dam et al., 2021). In total, 14 out of the 35 species-level clades in Cluster VI occurred only in marine sediments, while 11 occurred only in brackish sites, 9 were found across both marine and brackish sites, and one species-level clade (*Ca.* E. aarhusiensis) was found across marine, brackish and freshwater sediments (Table 4; Figure 4). While 32 of the 35 species-level clades in Cluster VI occurred in coastal sediments (<30 m depth), three were detected in deep sea sediments of >1,000 m depth. For instance, three sequences of one potential species originated from a deep sea methane cold seep in New Zealand (Ruff et al., 2013).

Overall, the species-level clades of Cluster VI also show substantial large morphological diversity, with cells of different species varying significantly in diameter and length (Figures 3C–E). *Ca.* E. rattekaaiensis, aestuarii/scaldis and antwerpensis are rather thin species, with reported diameters of *ca.* 1.2 μm, 0.7 to 1.2 μm and 0.4 to 0.5 μm, respectively. In contrast, *Ca.* E. gigas and laxa are the cable bacteria with the largest reported diameters, with diameter ranges from 2.0 to 8.0 and 1.0 to 6.0 μm, respectively.

## 4 Discussion

### 4.1 Attaining long consensus 16S rRNA gene sequences for phylogenetics

Long 16S rRNA gene sequences (≥800 bp) can be used to assign species-level taxonomies (White et al., 2010; Johnson et al., 2019) and attain a high phylogenetic resolution (Kim et al., 2011) of uncultured microbes like cable bacteria. Previously, full-length 16S rRNA gene sequences of cable bacteria have been extracted from (incomplete) genome assemblies of individual cable bacteria filaments after applying whole genome amplification (Risgaard-Petersen et al., 2015; Trojan et al., 2016; Kjeldsen et al., 2019; Geelhoed et al., 2023). More recent methods are full-length 16S PacBio amplicon sequencing of entire sediment communities (Xu et al., 2021; Sachs et al., 2022) or the extraction of 16S sequences from high-quality (meta)genomes obtained from sediment samples (Thorup et al., 2021; Sereika et al., 2023; Hiralal et al., 2024a; Hiralal et al., 2024b). These approaches however require extensive sequencing efforts. An alternative is the isolation of individual, or tufts of, filamentous bacteria from sediments, and the subsequent cell lysis and (RT-)PCR amplification for either clone libraries or direct sequencing, to obtain phylogenetic information of cable bacteria (Pfeffer et al., 2012; Schauer et al., 2014), as well as from other filamentous bacteria (Beggiatoaceae family) (Salman et al., 2011).

However, until now the direct 16S sequencing method has not yielded full-length 16S sequences (≥1,450 bp) of single cable bacteria filaments. The nested PCR method proposed here offers a number of advantages over previous methods. Firstly, applying nested PCR bypasses the need for clone libraries (Pfeffer et al., 2012; Schauer et al., 2014) or extraction and reamplification of amplicons from gel (Salman et al., 2011). Our approach furthermore provides high confidence and minimizes the risk of chimeric sequences by aligning multiple partial sequences from different regions of the 16S rRNA gene obtained by distinct primer pair combinations into consensus sequences. As such, the method consistently produced long (≥800 bp) to full-length (≥1,450 bp) 16S rRNA gene sequences, six of which represent new species-level clades.

To verify that filaments belonged to the cable bacteria, and also assess the morphological diversity of cable bacteria, hand-picked filaments were also imaged microscopically (Figure 3). Filaments showed long cell chains with characteristic continuous, longitudinal ridges, a distinct morphological feature typical for cable bacteria (Pfeffer et al., 2012; Malkin et al., 2014; Trojan et al., 2016; Cornelissen et al., 2018; Geelhoed et al., 2023). Yet, as for now, it is not possible to establish a direct link between phylogeny and morphology, as the same filament cannot be used for both AFM imaging and DNA sequencing. However, the sample of filaments that was imaged here does reveal a rather large morphological variation within the cable bacteria clade, both within as well as between different genus-level clusters (Figure 3).

Finally, one should be aware that sediments may contain different cable bacterium morphotypes at different densities (Marzocchi et al., 2018). Therefore, hand-picking of single filaments may induce a preferential retrieval of filaments that have a larger diameter and length, as well as strains that are relatively abundant. As a result, less data may be retrieved from filaments that have a low abundance, or which are thin and/or short, thus producing a bias in the dataset compiled. Despite this potential bias, the method proposed here offers a straightforward approach to obtain full-length 16S rRNA gene sequences, which also can be applied to other filamentous bacteria and those that are difficult to cultivate.

### 4.2 Extended diversity of cable bacteria

The updated phylogenetic tree of the cable bacteria clade reveals a substantially expanded diversity compared to previous assessments (Trojan et al., 2016; Geelhoed et al., 2023; Sereika et al., 2023; Hiralal et al., 2024a; Hiralal et al., 2024b; Plum-Jensen et al., 2024). Based on 16S rRNA gene comparison, we identified six genus-level clusters and 90 potential species-level clades (Figure 1). However, 38 of these species-level clades contained only a single sequence, while 52 clades contained multiple sequences. This pattern suggests that some cable bacterium species are far more widespread in the environment compared to others, so their chance of detection increases. In this view, the “single sequence clades” then represent a pool of rarely encountered cable bacterium species. Note however that some environments (e.g., river sediments, salt marshes) have been more frequently sampled, which may increase the presence of sequences and species-level clades tied to these environments. Moreover, some single sequence clades could potentially result from sequencing errors such as base substitutions, deletions, insertions or chimeras in metagenome-extracted or full-length amplicon 16S sequences (Schirmer et al., 2016; Delahaye and Nicolas, 2021). However, poor quality sequences were removed from the sequence alignment during data curation in the implemented quality control step.

The six genus-level clusters were primarily defined based on the grouping of species-level clades in the phylogenetic tree (Figure 1). Subsequently, we verified the 16S rRNA gene similarity of representative species sequences within and between genus-level clusters (Figure 2). All species-level clades of Clusters I, II, and III showed similarities of <94.0% compared to other clusters, thus respecting the traditional genus-level cutoff of 94.5% (Yarza et al., 2014). However, species within Clusters IV, V, and VI showed cross-cluster sequence identities of >94.5%, with a maximum of 95.9%, to each other (Figure 2), albeit their clearly separated placement in the phylogenetic tree (Figure 1). Additionally, the 16S rRNA gene sequence identity of the species within a respective cluster often dropped below the 94.5% genus-cutoff (Yarza et al., 2014), indicating a significant genetic divergence within cable bacteria. For instance, the minimum sequence identity within a cluster was observed in Cluster VI, where the species AUS1_2 and KX172796 were only 89.7% similar to each other.

The genus-level clustering is largely consistent with previous genus-level classifications originating from phylogenomic data (Geelhoed et al., 2023; Sereika et al., 2023). Three clusters can be directly linked to previously identified clades. Cluster VI corresponds to the *Ca.* Electrothrix genus, while Clusters IV and V, respectively, correspond to the potential AR4 and AR3 genera, which have been identified based on comparative genome analysis (Geelhoed et al., 2023), but which have not been named and described yet. A conspicuous observation is that the species that have been previously assigned to the *Ca.* Electronema genus are here divided over the two separate Clusters I and II. The reason for this is that they emerge as distinct clades within the phylogenetic tree (Figure 1). Moreover, the two most similar species between these clusters only show a 16S sequence identity of 92.0% (*Ca.* Electronema halotolerans vs. PP554486), which is well below the standard 94.5% genus-cutoff (Yarza et al., 2014). However, based on average nucleotide identity (ANI) and percentage of conserved proteins (POCP) the strain *Ca.* Electronema halotolerans has been placed in the *Ca.* Electronema genus (Sereika et al., 2023). This example illustrates the well-known limitation of using 16S phylogenetic information for the delineation of clades at the genus level (Hassler et al., 2022).

In addition to the known genus-level taxa, our dataset reveals one new genus-level clade (SI2) represented by Cluster III (Figure 1), which includes five sequences obtained from the Magazzolo river estuary (Sicily, Italy). Comparing the 16S sequences of SI2 with the representative sequences of the 89 other potential cable bacteria species identified here, provides a maximum sequence identity of 94.0% with *Ca.* Electrothrix (OBEP010014765), 92.3% with AR3, 91.8% with AR4 and 91.0% with *Ca.* Electronema (FQ658831) (Figure 2). SI2 may hence constitute a novel genus of cable bacteria, hinting at previously unexplored evolutionary diversity within the cable bacteria clade.

In order to delineate different bacteria species, a cutoff value of 98.7% 16S sequence identity is commonly used (Yarza et al., 2014). However, due to its conserved nature, the 16S rRNA gene cannot always accurately delineate closely related species (Větrovský and Baldrian, 2013; Lan et al., 2016). In order to examine if the 16S rRNA gene is suitable to delineate different cable bacteria species, sequence identities of the 13 described species were compared. Overall, the established species-level cutoff of 98.7% could be applied to successfully delineate most previously described cable bacteria species, but with two exceptions. Based on 16S data, *Ca.* En. aureum and *Ca.* En. nielsenii should be designated as one species (99.4% sequence identity). Likewise, *Ca.* E. laxa falls within the species-level range of *Ca.* E. communis and *Ca.* E. aarhusiensis (98.9 and 98.8%, respectively) (Figure 2). However, average nucleotide identities (ANI) of <94% of the available genomes revealed that these are indeed separate species based on the 95% ANI threshold (Jain et al., 2018; Sereika et al., 2023).

By definition, 16S rRNA gene sequences contain less information than whole genomes, with phylogenomic trees providing higher taxonomic resolution than 16S phylogenetic trees. This is known to potentially cause differences in the tree topology (Ankenbrand and Keller, 2016; Hug et al., 2016). Still, comparing our 16S rRNA gene tree with previously published phylogenomic trees (Geelhoed et al., 2023; Sereika et al., 2023; Hiralal et al., 2024a; Hiralal et al., 2024b; Plum-Jensen et al., 2024), there appears only one major difference in topology. In the 16S tree, the AR3 genus (Cluster V) is placed between the AR4 genus (Cluster IV) and *Ca.* Electrothrix (Cluster VI), while in recent genome trees, AR3 is placed between AR4 and *Ca.* Electronema (Clusters I/II). This different placement may be due to poor bootstrap support of the AR3 and AR4 clades (Figure 1), as also seen in previous 16S rRNA gene trees (Dam et al., 2021; Geelhoed et al., 2023; Plum-Jensen et al., 2024), or may result from the expanded number of sequences adjacent to AR3 in Cluster V. The assignment of bacterial genus-level clades is conventionally based on genomic information—a commonly used threshold for genus classification is 65% amino acid identity (AAI) (Konstantinidis et al., 2017). At present, high quality (unfragmented) genomes are only available from three clusters (Clusters I, II, and VI). Thus additional, high-quality genomes are needed from Clusters III, IV, and V to obtain a more accurate resolution of the taxonomy of the cable bacteria. Providing whole genomes is also an essential requirement to describe and name any of the new species proposed here.

An important question is whether cable bacteria truly comprise a monophyletic clade. Currently, all sequences attributed to cable bacteria belong to one monophyletic clade, apart from one possible exception. Putative cable bacteria detected in aquifers form a genetically distinct sister clade to *Desulfurivibrio* spp. within the Desulfobacterota that does not fall within the 6-cluster clade in the Desulfobulbaceae family examined here (Müller et al., 2015; Müller et al., 2020; Sachs et al., 2022). These aquifer strains appear to be physiologically distinct from the known Electrothrix/Electronema strains, as a they have a single cell stage and are capable of sulfur disproportionation (Müller et al., 2015; Müller et al., 2020). It should be noted that the approach taken here cannot resolve whether the cable bacteria clade is monophyletic or not, as both the primers used in the nested PCR as well as the database search only targets sequences within the Desulfobulbaceae family. At present, little microscopic, electrochemical and/or spectroscopic data are available for the aquifer strains, and hence it is not clear whether these organisms are truly capable of long-range electron transport and electrogenic sulfide oxidation. This is also the case for many of the potential cable bacteria sequences obtained from public databases for which no metabolic or geochemical data is available. The capacity for long-range electron transport can be assessed in a number of ways: either directly by measuring the current through filaments (Meysman et al., 2019; Bonné et al., 2020; Pankratov et al., 2024) or indirectly, by microscopy or spectroscopy. Detailed microscopy can document the unique topography of their cell surface, which reveals a series of ridges that run in parallel along the full length of the bacterial filament, and which each embed a single fiber (*ca.* 50 nm diameter) that carries the electron current (Cornelissen et al., 2018; Meysman et al., 2019). Detailed spectroscopy can document the Raman fingerprint of cable bacteria, which originates from the Ni cofactor involved in long-range electron transport, and hence is highly specific and unique to cable bacteria (Boschker et al., 2021; Smets et al., 2024). Hence, in future studies, when describing and naming new bacterial clades, it is highly recommended to explicitly assess these physiological, microscopic and spectroscopic features that are unique to cable bacteria (Hiralal et al., 2024a; Hiralal et al., 2024b).

### 4.3 Environmental adaptation patterns in cable bacteria phylogeny

Cable bacteria belong to the Desulfobulbaceae family, which was recently taxonomically reclassified and currently encompasses the genera *Desulfobulbus*, *Desulfogranum*, *Desulfolithobacter*, *Ca.* Electrothrix, and *Ca.* Electronema (Waite et al., 2020; Hashimoto et al., 2022). They were suggested to have evolved from a group of strictly anaerobic, sulfate-reducing bacteria (Kjeldsen et al., 2019), although their exact evolutionary origin, and whether they evolved from a saltwater or freshwater environment, remains uncertain. Recently, molecular clock estimates suggested that cable bacteria may have diverged *ca.* 0.56 billion years ago, either during or after the Neoproterozoic oxygenation event (Cui et al., 2024).

A marine origin of cable bacteria was suggested previously (Klier et al., 2018; Dam et al., 2021), and in this view, the ability of long-distance electron transport evolved once in the Desulfobulbaceae family before a divergence into a freshwater and a marine lineage took place (Risgaard-Petersen et al., 2015). The recently described species *Ca.* En. halotolerans has been suggested to be an evolutionary link between the two previously described genera *Ca.* Electrothrix and *Ca.* Electronema (Sereika et al., 2023). Additionally, the *ca.* 1 Mbp smaller genome in *Ca.* En. aureum compared to *Ca.* Electrothrix species suggests a loss rather than a gain of osmoregulation genes (Na^+^/H^+^ antiporter *NhaA*) required for life in saltwater environments (Kjeldsen et al., 2019; Dam et al., 2021; Sereika et al., 2023). Our results here support this hypothesis. The majority of the species (7 of 11 species) of the genera *Desulfobulbus*, *Desulfogranum* and *Desulfolithobacter* as well as 84% of the cable bacteria species-level clades that we identified (76 of 90), originate from brackish/marine environments (Figure 4B). Together these findings indeed suggest a longer evolutionary history of cable bacteria in saltwater environments and a later occupation of freshwater sediments.

We propose that the branching of Clusters I, II, and III could be linked to a freshwater-based adaptation (Figure 1). Cluster I encompasses species exclusively from genuine freshwater environments. This is in concordance with the results of a recent survey, in which no *Ca.* Electronema 16S sequences (excluding *Ca.* En. halotolerans) were detected at salinities higher than 0.3 (Dam et al., 2021). Sequences from Cluster I also never co-occur with (salt-tolerant) sequences from other clusters, thus lending support to a specific restriction to freshwater environments and dedicated metabolic adaptations that prevents them from thriving at brackish and marine salinities (Kjeldsen et al., 2019; Dam et al., 2021; Sereika et al., 2023). In contrast, sequences in Clusters II and III have a “brackish” signature, as they are retrieved from sediments in estuaries, which had intermediate salinities (range 2–20) at the time of sampling and during incubations (sites: ME2, ME3, ME20, and YR17, Table 1). Estuaries are dynamic systems, with spatial and temporal fluctuations of environmental parameters such as salinity, temperature, sediment grain size and oxygen availability (Borsuk et al., 2001; Cloern et al., 2017), as well as temporal variation in microbial community composition (Bernhard et al., 2005; Zheng et al., 2014). In addition to the *Ca.* En. halotolerans, we identified three novel species-level clades (Cluster II: SI1 and YR; Cluster III: SI2) from sites in the Magazzolo and Yarra River estuaries (Table 4). These two sites are known to exhibit strong salinity fluctuations (Roberts et al., 2012) (personal observations). Therefore, one hypothesis could be that Clusters II and III are not so much adapted to steady “brackish” conditions, but have specific adaptions to cope with strong and fast salinity fluctuations.

Clusters IV, V, and VI have a “marine” signature as they all contain sequences retrieved from sediments with full marine conditions (salinity ≥30). However, a high salinity does not seem to be a necessary requirement. For instance, within Cluster VI, species *Ca.* E. aarhusiensis and communis can survive at a wide range of salinities (0.3–35, and 0.3–21, respectively) (Trojan et al., 2016; Dam et al., 2021). *Ca.* E. aarhusiensis was also found in a freshwater lake in Denmark (Karst et al., 2018), whereas our data extends the salinity tolerance of *Ca.* E. communis to 32 (BCZ130). Moreover, *Ca.* Electrothrix clades SI3 and EB2 (Cluster VI) were detected at lower salinities (S = 2 and 10, respectively) than *Ca.* En. halotolerans and clades SI1 and YR (Cluster II). The detection of EB2 at salinities 10 and 33 suggests a potential broad salinity tolerance of this clade, from brackish to marine systems. The specific physiological adaptations of the “brackish” Cluster groups II-III versus the “marine” Cluster groups IV-VI require further elucidation. As noted above, the absolute salinity level may not be the only important factor, but also the particular temporal pattern of salinity fluctuations may play a role.

Interestingly, sequences within Cluster V were mainly retrieved from deep sea environments. In fact, 28 of the 35 identified species-level clades in Cluster V originate from sites deeper than 700 m, across geographically distant areas, including the Gulf of Mexico, the Atlantic, the Indian and the Pacific Ocean (Figure 4A; Supplementary Table S5). While deep-sea strains are present in Clusters IV and VI, they are less prominent: one species-level clade in Cluster IV was retrieved from deep sea sediments, and three species-level clades from Cluster VI. This “deep sea” signature of Cluster V is remarkable, as cable bacteria have been predominantly associated with shallow or coastal environments, which typically have shown high sulfate reduction and hence high sulfide production rates. Cluster V species have mostly been retrieved from sites near hydrothermal vents and cold seeps (Grünke et al., 2011; Ruff et al., 2013), which are local hotspots of sulfide production in the deep sea, which hence enables electrogenic sulfur oxidation. In comparison to other deep sea environments, these sites are likely overrepresented. Nonetheless, the cable bacteria from Cluster V represent an enigmatic group that could be specifically adapted to deep sea sulfidic environments. Such a separation of lineages with water depth (deep sea versus coastal environments) was observed for the gammaproteobacterial order *Woeseiales*, which occurs globally in marine surface sediments (Hoffmann et al., 2020).

### 4.4 Coexistence of different cable bacteria taxa

Our data indicate that coexistence of multiple cable bacteria species is possible in diverse habitats from freshwater lakes to deep sea sediments. Different morphologies of cable bacteria (e.g., filaments with varying diameters) are frequently observed in the same sediment sample (Scholz et al., 2021; Geelhoed et al., 2023), thus suggesting strain diversity. Direct evidence of coexistence of different *Ca.* Electrothrix species was first reported for a Baltic Sea site by fluorescence *in situ* hybridization (Marzocchi et al., 2018). Cable bacteria amplicon sequences (*ca.* 400 bp) from multiple clusters (IV, VI, VI) were detected in the same sediment near fish farms in Iceland (Vasquez-Cardenas et al., 2022), filaments belonging to multiple clusters (IV, V, VI) and to multiple species within the *Ca.* Electrothrix cluster (VI) were detected at marine or brackish sites by whole genome sequencing (Geelhoed et al., 2023) and two *Ca.* Electronema species were conjointly detected in a freshwater sediment in California (Yang et al., 2023).

Our survey here indicates that coexistence of multiple cable bacteria species is very common. Our dataset of 16S rRNA gene sequences includes 74 sampling sites across the world with sequences longer than 800 bp (Table 3; Figure 4). Seven out of these 74 sites harbored sequences from more than one genus-level cluster, whereas half of the sites contained two or more species-level clades. Ten sites showed an even higher diversity with 4 to 12 species species-level clades being present (Figure 4C; Table 5; Supplementary Table S5).

**Table 5:** Sites with at least two clusters or four species-level clades of cable bacteria with at least one sequence ≥800 bp.

Site	Habitat	Clusters	# species	References
Mound 12, Costa Rica	Deep sea	IV, V	12	Thurber et al. (2011) and Dekas et al. (2016)
RSM30	Intertidal	VI	9	This study; Geelhoed et al. (2023), Hiralal et al. (2024a), Hiralal et al. (2024b), Plum-Jensen et al. (2024)
BCZ130	Subtidal	IV, V, VI	7	This study; Geelhoed et al. (2023)
Aarhus Bay	Subtidal	VI	6	Pfeffer et al. (2012) and Trojan et al. (2016)
Fano, Denmark	Intertidal	V	5	Karst et al. (2018)
Mariagerfjord, Denmark	Fjord	V	5	Karst et al. (2018)
Ulvedybet, Limfjorden, Denmark	Fjord	VI	5	Karst et al. (2018)
Wenyu river, China	River	I	5	Xu et al. (2021)
Hydrate Ridge, Oregon	Deep sea	V	4	Knittel et al. (2003) and Marlow et al. (2014)
YR	Estuary	II, VI	4	This study; Geelhoed et al. (2023)
ME2	Estuary	II, III, VI	3	This study
Loegten strand, Denmark	Coastal	II, VI	3	Geelhoed et al. (2023) and Sereika et al. (2023)
Mississippi Canyon Block 118, Gulf of Mexico	Subtidal	IV, V	2	Lloyd et al. (2010)
Hikurangi margin, Wairarapa - Takahae	Deep sea	V, VI	2	Ruff et al. (2013)

The question as to how several species with apparently the same metabolic requirements coexist thus arises. It was previously proposed that higher electron donor availability, in freshwater sediments, may reduce inter-species competition among cable bacteria species, thus allowing less dominant species to co-occur (Xu et al., 2022). This could explain why organic-rich and high-sulfide sites (RSM30, Aarhus Bay and YR17) harbor multiple cable bacteria species (Trojan et al., 2016; Geelhoed et al., 2023). Conversely, our analysis suggests that a shortage of the electron donor sulfide may also stimulate diversity. Effectively, there were two sites (BCZ130 and ME2) that harbored species-level clades from three different clusters, and these two sites showed low free sulfide concentrations (Table 5; Supplementary Table S5). Cryptic sulfur cycling, i.e., simultaneous reduction of sulfate and oxidation of sulfide, may be taking place in these sediments which can prevent the accumulation of sulfide in the porewater (Van de Velde et al., 2016; Burdorf et al., 2017). Cable bacteria species inhabiting these low-sulfide sediments may have a high affinity for sulfide (Meysman et al., 2015), and hence, sulfide availability could be a factor driving diversity (in addition to salinity). Different substrate affinities between species can lead to micro-niche differentiation, as seen for different coexisting *Achromatium* species, which show a shift in relative abundances in anoxic slurry incubations compared to oxic ones (Gray et al., 2004). Indeed, we found no overlap between species inhabiting non-sulfidic sediments compared to sulfidic ones, except for *Ca.* E. communis, which could indicate species-specific adaptations to different levels of sulfide. It should be noted that only few studies explicitly reported H_2_S concentrations of investigated sediments, which impedes conclusions about the effect of H_2_S on cable bacteria species compositions. Ten sites showed sulfide concentrations of <10 μM. Six of these were brackish sites, incubated in this study (ME2, ME3, ME20, ED33, BCZ130, and BCZ700), which interestingly revealed potential novel or undescribed cable bacteria taxa from clusters II (SI1, SI2), IV (AR4), and VI (SI3) (Table 4).

Overall, the relation between coexistence and niche differentiation remains to be further examined for cable bacteria. Presently, it is unknown which environmental conditions facilitate or impede the coexistence of cable bacteria and how different cable bacteria species might interact or compete with each other. Targeted time series sediment manipulation with treatments such as different electron donor (Xu et al., 2021) and acceptor availabilities may further illuminate the conditions selecting for a dominant species of cable bacteria or the coexistence of multiple species.

### 4.5 Habitats and species distribution

Cable bacteria 16S rRNA gene sequences (≥800 bp) were compiled from 92 different sampling sites across the world (Table 3; Figure 4A). More than half of the locations (*n* = 53) were coastal sites (≤200 m water depth), 23 were deep sea locations (1,000–3,843 m) and 16 were freshwater sites (Figure 4B). These sites include a broad range of temperatures in incubations with subpolar to tropical sediments from 0 to 25°C, indicating a broad temperature tolerance (Burdorf et al., 2017). The absence of long cable bacteria 16S sequences in African, South American and (sub)polar locations in the present dataset is likely due to the lack of studies investigating surface sediments in these geographical regions (Figure 4A).

Coastal sites encompassed a diverse array of habitats, including mud flats and mud banks, (intertidal) salt marshes, seagrass beds, mangroves, bivalve reefs, seasonally hypoxic basins, marine lakes and estuaries (Malkin et al., 2014; Burdorf et al., 2017; Dam et al., 2021; Scholz et al., 2021; Geelhoed et al., 2023). Deep sea habitats included hydrothermal vents, mud volcanoes and cold seeps (Grünke et al., 2011; Ruff et al., 2013; Vigneron et al., 2014) and some cable bacteria sequences even originated from the surfaces of deep sea fauna, such as gastropods and yeti crabs (Goffredi et al., 2004; Thurber et al., 2011). Freshwater sites constitute lakes, streams and aquifers (Trojan et al., 2016; Sachs et al., 2022), and one potential *Ca.* Electronema (Cluster I) sequence was detected in a prairie soil in Canada (MG394614), thus introducing terrestrial environments as a possible cable bacteria habitat (Figure 4B).

When zooming into the geographic distribution of species-level clades, two patterns emerge: global versus local distributions (Figure 4A; Supplementary Table S4). *Ca.* E. gigas (Cluster V) shows a global distribution as it was found in intertidal salt marsh sediments in the Netherlands (S ∼ 30), a brackish sediment in Denmark (S ∼ 18–23) and an estuary with high salinity fluctuations (5–30) in Australia (Burdorf et al., 2017; Geelhoed et al., 2023; Sereika et al., 2023). High sulfide-tolerance may be a species-specific characteristic of *Ca.* E. gigas as all three locations are sulfide-rich coastal sediments (Geelhoed et al., 2023). Likewise, *Ca.* En. palustre and nielsenii (Cluster I) were found across geographically distant locations, from a stream and an artificial pond in Denmark to rivers in both southern Germany and eastern China (Trojan et al., 2016; Xu et al., 2021; Sachs et al., 2022). *Ca.* E. communis also appears to be a generalist clade, occupying a broad ecological niche (BCZ130, RSM30, Aarhus Bay, Little Sippewissett salt marsh, MA, United States), coping with a wide range of salinities and free sulfide concentrations (from below detection limit to >1,000 μM), rather than being specifically adapted to environmental conditions of the Baltic Sea as has been suggested previously (Dam et al., 2021).

In contrast, certain species-level clades (such as SI1, SI2, SI3, EB2, and YR) show an “endemic” occurrence, being only detected at a single site (Supplementary Table S4). In total, 63 out of 98 potential species-level clades were found at a single site, including 31 assigned to Cluster V which mostly originate from deep locations of ≥700 m depth (Table 4; Figure 4A). This high number of potential “endemic” species indicates that a link between the phylogeny and the geographical distribution of cable bacteria may exist and that some cable bacteria species may have evolved site-specific adaptations. Further screening of aquatic sediments is however needed to confirm the “endemic” biogeographic character of certain strains.

## Data Availability

Near full-length 16S rRNA gene sequences can be accessed on GenBank under accession numbers PQ214937 to PQ214942. Shorter species-level clade sequences have accession numbers PQ216432 and PQ216433.

## Footnotes

^1^ https://github.com/tseemann/barrnap
