# Supplementary material for: On the diversity, phylogeny and biogeography of cable bacteria
Source: Front Microbiol. 2024 Nov 19;15:1485281. doi: 10.3389/fmicb.2024.1485281 (PMC11611824; doi:10.3389/fmicb.2024.1485281)
Supplement: Supplementary file 1 [file Table_1.DOCX]

Supplementary Material

# Supplementary Methods

**Sampling site descriptions**

**Belgian Coastal Zone:**

Both subtidal sites BCZ130 and BCZ700 are located ca. 5 km off the Belgian coast in the North Sea and had a salinity of 30. The sediment mainly consist of cohesive clay and mud. While bioturbating fauna is often absent from BCZ130, BCZ700 is inhabited by brittle stars.

Station BCZOH is located inside the harbor of Ostend and consisted of fine-grained homogeneous, black, sulfidic mud (Zhou et al., 2020).

**Ebro salt marsh:**

Two locations were selected in a salt marsh in the Ebro delta in Spain. Site EB10 was a small pond with approximately 30 cm of overlaying water at a salinity of 10.

Site EB33 was located in a different pond in the salt marsh with a higher salinity of 33 and contained dark-grey colored, sandier sediment.

**Magazzolo estuary:**

The Magazzolo estuary is located in Seccagrande in Sicily, Italy, where three locations were selected. Sampling location SI2 was a closed lagoon (salinity 2), separated by a sand bank from the Mediterranean Sea. The sediment consisted of grey clay. SI3 (salinity 3) was located at the right bank of the Magazzolo river approximately 350 meters land inward. The sediment came from between cobble stones and bulrush roots. Sediment was colored light grey and appeared as clay mixed with some sand. SI20 (salinity 20) was a smaller isolated lagoon on the left bank of the river Magazzolo. Due to a sand bank, no connection to the river or the Mediterranean Sea and no inflow or outflow was apparent. The sediment had a dark grey color.

**Rattekaai salt marsh:**

The Rattekaai salt marsh contains multiple intertidal creeks with fine-grained sediment that contains high organic matter concentrations and is rich in sulfide. The salinity is approximately 30. No bioturbating animals are typically present (Malkin et al., 2014).

**Yarra River estuary:**

The Yarra river estuary is located close to Scotch College in Melbourne, Australia. The sampling site contains a salt wedge and the sulfidic sediment turns hypoxic periodically. Because of seasonally low rainfall, the site undergoes high salinity fluctuation ranging from 2 to 38 (Roberts et al., 2012) .

**References**

Malkin, S.Y., Rao, A.M., Seitaj, D., Vasquez-Cardenas, D., Zetsche, E.M., Hidalgo-Martinez, S., et al. (2014). Natural occurrence of microbial sulphur oxidation by long-range electron transport in the seafloor. *ISME J* 8(9)**,** 1843-1854. doi: 10.1038/ismej.2014.41.

Roberts, K.L., Eate, V.M., Eyre, B.D., Holland, D.P., and Cook, P.L.M. (2012). Hypoxic events stimulate nitrogen recycling in a shallow salt-wedge estuary: The Yarra River estuary, Australia. *Limnology and Oceanography* 57(5)**,** 1427-1442. doi: 10.4319/lo.2012.57.5.1427.

Zhou, C., Gaulier, C., Luo, M., Guo, W., Baeyens, W., and Gao, Y. (2020). Fine scale measurements in Belgian coastal sediments reveal different mobilization mechanisms for cationic trace metals and oxyanions. *Environ Int* 145**,** 106140. doi: 10.1016/j.envint.2020.106140.

# Supplementary Figures and Tables

For more information on Supplementary Material and for details on the different file types accepted, please see [here](https://www.frontiersin.org/guidelines/author-guidelines#supplementary-material).

## Supplementary Figures

**
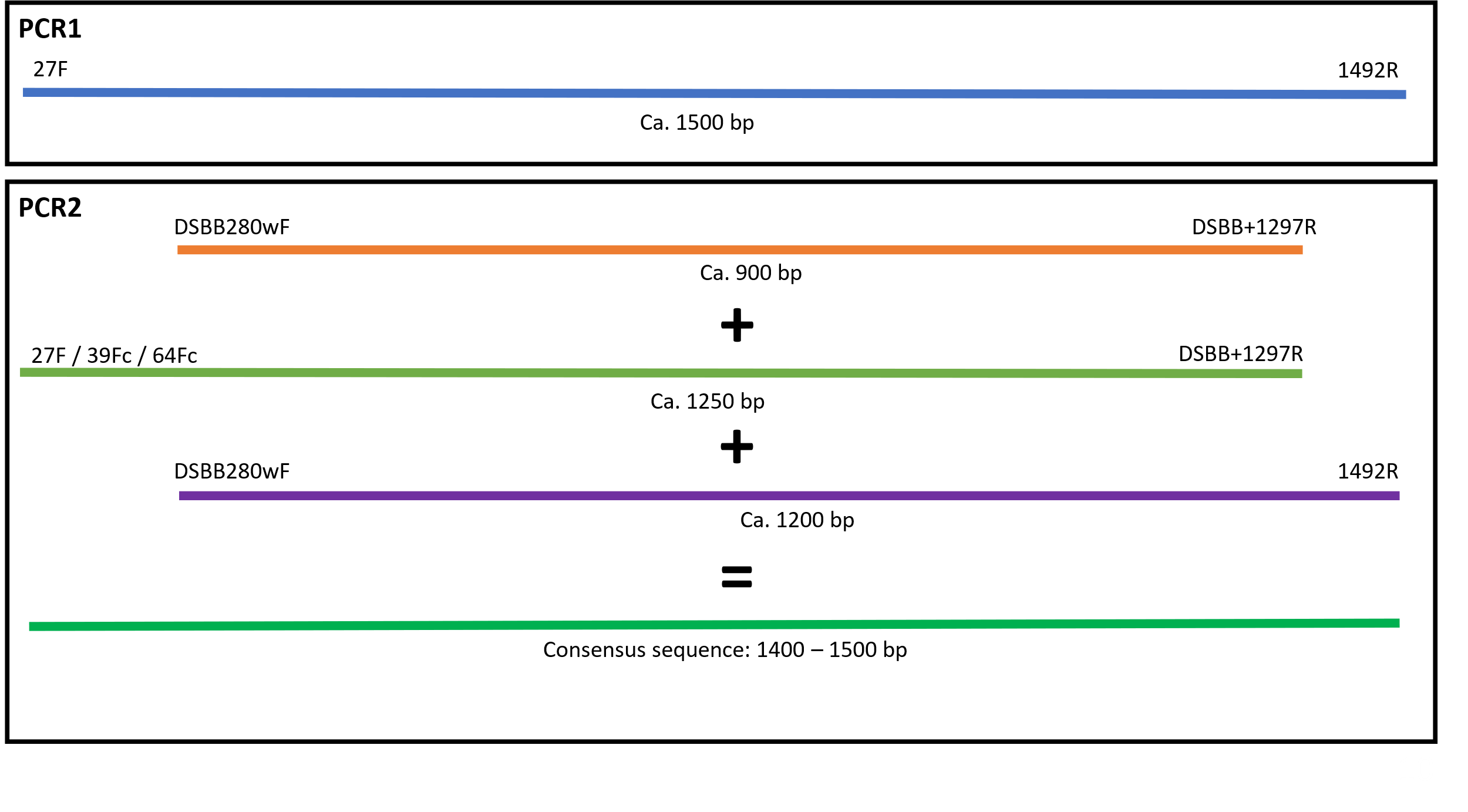
**

**Supplementary Figure 1.** Illustration of the single filament nested PCR method. In PCR1, the entire 16S rRNA gene is amplified with universal primers, before different regions of the PCR1 product are amplified using a combination of universal and Desulfobulbaceae-specific primers, yielding near full-length 16S rRNA gene sequences (≥1400 bp).

**
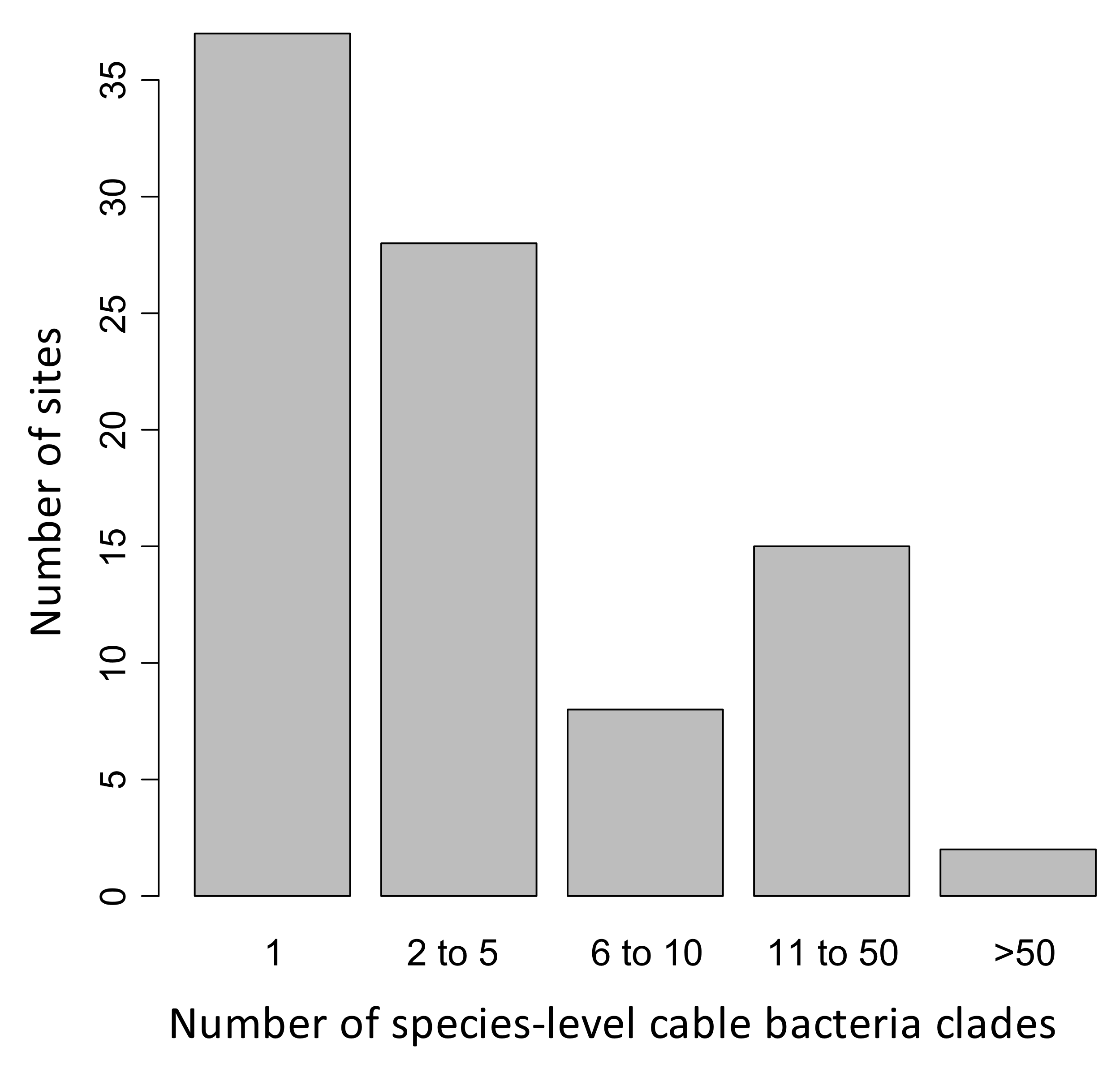
**

**Supplementary Figure 2.** Bar plot indicating the number of species-level cable bacteria clades (≥800 bp) present at the number of different sites.


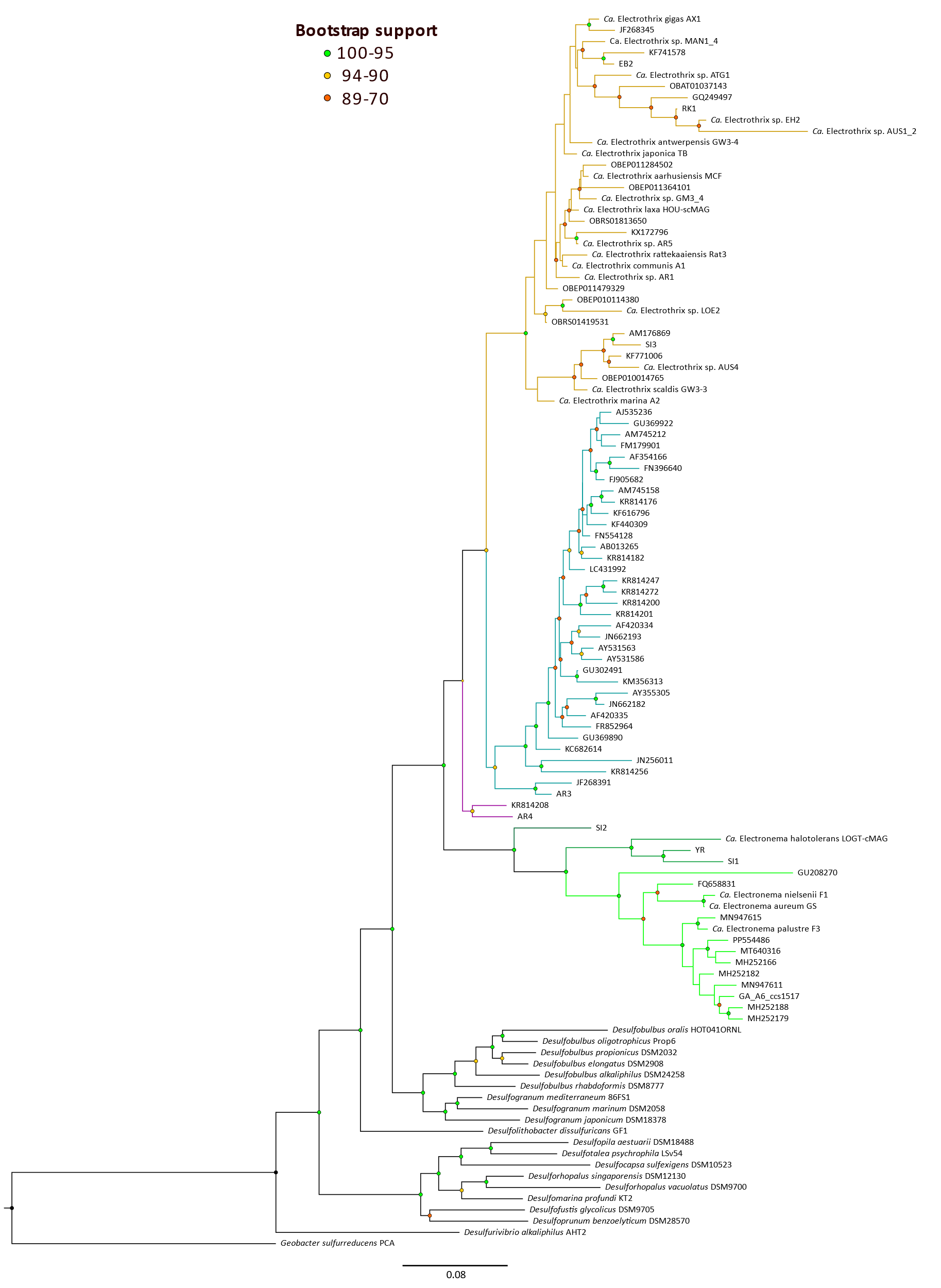


**Supplementary Figure 3.** Rectangular view of 16S rRNA gene phylogenetic tree of the six clusters and 90 cable bacteria species-level clades from Figure 1. Colored circles/nodes indicate bootstrap support (1000 iterations) and colored branches indicate clusters. The tree was run in IQ-TREE v1.6.12 in ultrafast mode with best-fit model TIM3e+I+G4. All sequences used are listed in Supplementary Table 4.
